# Retinoic acid receptor β deletion in podocytes causes kidney and liver dysfunction, modeling nephrotic syndrome

**DOI:** 10.1530/JME-25-0146

**Published:** 2026-01-02

**Authors:** Yuling Chi, Krysta M DiKun, Xiao-Han Tang, Charles D Warren, Shireen Chikara, Eduardo Mere Del Aguila, John A Wagner, Jacob B Geri, Lorraine J Gudas

**Affiliations:** ^1^ Pharmacology Department, Weill Cornell Medicine of Cornell University, New York, New York, USA; ^2^ Brain and Mind Research Institute, Weill Cornell Medicine of Cornell University, New York, New York, USA; ^3^ Cell and Developmental Biology, Weill Cornell Medicine of Cornell University, New York, New York, USA; ^4^ Department of Medicine, Weill Cornell Medicine of Cornell University, New York, New York, USA

**Keywords:** retinoic acid receptor, nephrotic syndrome, albuminuria, podocyte, lipid, liver

## Abstract

Differentially altered expression of transcripts of retinoic acid receptors α, β, γ (*Rarα*, *β, γ*), which mediate the actions of all-trans retinoic acid (RA), is observed in glomeruli of nephrotic syndrome (NS) patients vs normal individuals, with *Rarβ* reduced and both *RARα* and *RARγ* increased. Thus, we generated a mouse model (PCRB) with *Rarβ* specifically deleted in podocytes to define the glomerular actions of *Rarβ*. *Rarβ* deletion in PCRB mice results in podocyte loss, podocyte foot process effacement, glomerular basement membrane (GBM) thickening, reduced podocyte adhesion to the GBM, lipid accumulation in glomeruli, and hyperfiltration leading to albuminuria. Genome-wide transcriptomics and proteomics studies of glomeruli revealed that *Rarβ* deletion increased *Mogat, Dgat*, and *Hmgcs* mRNAs, which catalyze triglyceride and cholesterol synthesis, and *Slc27a2* and *Cd36*, which mediate fatty acid uptake, recapitulating NS symptoms. Surprisingly, podocyte-specific *Rarβ* deletion also increased key mRNAs and proteins involved in fatty acid uptake and lipid biosynthesis in the liver, promoting steatohepatitis and systemic hyperlipidemia. These data indicate that *Rarβ* signaling in the kidney has a profound impact on both kidney and liver functions and suggest that *Rarβ* plays an important role in regulating kidney-liver crosstalk. PCRB mice may be a useful model of NS.

## Introduction

Nephrotic syndrome (NS) is one of the major causes of advanced kidney disease (Verma & Patil 2024). Clinical features of NS include edema, proteinuria, hypoalbuminemia (abnormally low levels of albumin in the blood), hypertension, hyperlipidemia, and hypercoagulation. NS is divided into 3–4 main types: minimal change disease (MCD), focal segmental glomerulosclerosis (FSGS), membranous nephropathy (MN), and others (O) (Noone *et al.* 2018, Barutta *et al.* 2022, Verma & Patil 2024). NS in adults is often caused by focal segmental glomerulosclerosis (FSGS), diabetic nephropathy, or MN (Barutta *et al.* 2022). Idiopathic NS also occurs in children (Noone *et al.* 2018).

NS is one type of glomerular disease, a common cause of chronic kidney disease, which represents a major global public health problem (Jha *et al.* 2013). Glomeruli contain three major cell types: endothelial cells, mesangial cells, and podocytes. Podocytes are epithelial cells in the glomerulus of the kidney that send out processes that, once mature, become foot processes attaching to glomerular capillaries (Garg 2018). The glomerular filtration barrier is comprised of three components: the glomerular endothelium, the basement membrane, and the slit diaphragms, specialized cell–cell tight junctions formed by these interdigitating podocyte foot processes. The slit diaphragms are the main barriers that keep proteins from moving into the urine. Nephrin, a major protein in podocytes, is located at the slit diaphragm (Ruotsalainen *et al.* 1999). Various types of injury to podocytes result in effacement, an early structural change in the contractile actin cytoskeleton of podocyte foot processes, and later in loss of their filtration barrier function, leading to abnormally high levels of protein, including albumin, in the urine (albuminuria) (Nagata 2016). Currently available treatments for NS are limited, and many patients do not respond to these treatments (Politano *et al.* 2020).

Retinoids, including various metabolites and derivatives of vitamin A (retinol), preserved renal function, decreased albuminuria, and reduced glomerular and tubular damage in rat models of acute and chronic mesangioproliferative glomerular nephropathy (Lehrke *et al.* 2002). Moreover, retinoids prevented podocyte injury and proteinuria in a rat model of puromycin-induced nephrosis (Moreno-Manzano *et al.* 2003). All-trans retinoic acid (RA), an active metabolite of vitamin A, exerts its biological signals and transcriptional changes via binding to retinoic acid receptor proteins (RARs) (Gudas 2022*a*). Our laboratory and others have demonstrated that RA and a RARβ2-selective agonist, AC261066, can attenuate symptoms of diabetic nephropathy by reducing albuminuria and glomerulosclerosis (Sierra-Mondragon *et al.* 2018, Trasino *et al.* 2018, DiKun & Gudas 2023). Our laboratory also demonstrated the protective effects of AC261066 in murine models of metabolic syndrome; AC261066 restored glycemic control, reduced pancreatic β cell mass and insulin secretion, and attenuated renal fibrosis (Trasino *et al.* 2016, Trasino *et al.* 2018). However, the detailed molecular mechanisms by which retinoids and the RARs mediate these biological and potentially useful clinical effects in the kidney are largely unknown.

We conducted a data search and obtained a database of genome-wide mRNA sequencing of glomeruli from normal individuals and NS patients provided by the Nephrotic Syndrome Study Network (NEPTUNE) and deposited in Gene Expression Omnibus (GSE197307) (Fan *et al.* 2025). Our data analysis indicated that the transcripts of all three *RARs* are detectable and are differentially altered in glomeruli from NS patients compared to normal individuals. *Rarβ* mRNA is the most abundant one among the three. *Rarβ* is significantly reduced, whereas both *Rarα* and *Rarγ* mRNAs are increased in NS. These data led us to hypothesize that reduced *Rarβ* levels could contribute to NS development. To test this hypothesis, we generated a mouse model with the *Rarβ* gene specifically deleted only in podocytes. We show that *Rarβ* expression in podocytes is required for normal glomerular morphology, gene expression, and function. Moreover, we show that podocyte-specific deletion of *Rarβ* results in NS, with lipid accumulation not only in glomeruli but also in the liver, and increased serum triglyceride (TG) and cholesterol levels.

## Materials and methods

### Acquisition and analysis of mRNA-seq data from NS patients and normal individuals

We acquired a database (GSE197307) of glomerular transcriptome for the Nephrotic Syndrome Study Network (NEPTUNE) cohort from The National Center for Biotechnology Information (NCBI). Detailed information on the database and analysis output is provided in the Supplementary Materials (see section on Supplementary materials given at the end of the article).

### Mice and treatments

All animal experiments and protocols were approved by the Institutional Animal Care and Use Committees (IACUC). A transgenic mouse line (PCR) expressing Cre under an *Nphs2* (podocin) promoter (Pod-Cre), obtained from Jackson Labs (JAX stock #008205, C57Bl/6 background) (Moeller *et al.* 2003), was crossed with floxed *Rarβ* mice (C57Bl/6 background), which resulted in a line with *Rarβ* specifically deleted in podocytes (PCRB) (Chapellier *et al.* 2002).

### Genotyping

The transgenic mice were genotyped using the primers listed (Supplementary Table 1) (Chi *et al.* 2025*a*).

### qRT-PCR

Reverse transcription and quantitative PCR were conducted as described (Melis *et al.* 2023).

### Assessment of renal and liver functions

Renal functions were assessed as described (Chi *et al.* 2025*b*). Liver functions were assessed by measuring blood serum ALT (alanine transaminase), AST (aspartate transaminase), TG, and cholesterol by the Laboratory of Comparative Pathology, Memorial Sloan Kettering.

### Immunostaining

Immunohistochemistry (IHC) was conducted as described (Chi *et al.* 2025*a*). The sources and dilutions of antibodies are listed (Supplementary Table 2). Immunofluorescence staining with Bodipy was as described (Laursen *et al.* 2021).

### Isolation of glomeruli

We isolated glomeruli based on a published procedure (Wang *et al.* 2019) with some modifications. Two fresh kidneys were harvested from a newly sacrificed mouse and each kidney was cut into two pieces. Cortices were collected and minced in an Eppendorf tube containing 500 μL of 1× HBSS (VWR; 02-0121-0500), and centrifuged at 290 *g*, 4°C, for 1 min. The supernatants were discarded, and pellets were transferred into a 15 mL tube containing 4 mL of digestion buffer, 1× HBSS with Collagenase Type V (Sigma, USA; C9263) at a concentration of 1 mg/mL, and digested in a 37°C water bath for 15 min, during which the digestion mixture was mixed by pipetting ten times at 5 min intervals. Digestion was stopped by adding 5 mL of Dulbecco’s modified Eagle’s medium (MP Biomedicals, USA; 1033122) containing 10% fetal bovine serum (FBS) (R&D Systems, USA; S11150H), and centrifuged at 290 *g*, 4°C, for 1 min. Supernatants were discarded, and pellets were resuspended in 5 mL cold HBSS and gently mixed by pipetting ten times. The mixtures were sieved through a pre-wetted 100 μm mesh strainer. Residual tissue on the strainer was pressed with the plunger of a 3 mL syringe. The 15 mL tube was then rinsed three times with 2, 2, and 1 mL cold HBSS, each time sieving through the 100 μm strainer. The filtrate was then sieved through a pre-wetted 70 μm mesh strainer and washed with an additional 35 mL of cold HBSS. The filtrate (∼45 mL) was collected and sieved through a pre-wetted 40 μm strainer. All glomeruli were on the top side of the 40 μm strainer. We flipped the 40 μm strainer upside down into a 10 cm plate and washed the contents onto the plate with 10 mL cold HBSS. The plates were placed on ice for 1–2 min to allow the tubules to attach. The floating glomeruli were transferred to a 50 mL centrifuge tube. The plate was rinsed twice with 10 mL cold HBSS, and the HBSS was transferred to the same 50 mL tube. We then added 0.01% (g/mL) polyvinylpyrrolidone (ThermoScientific, USA; J62417.22) and gently flipped the tube up and down twice. After centrifugation at 290 *g*, 4°C, for 3 min, the supernatant was discarded, and the glomeruli were resuspended in 100 μL HBSS.

### RNA extraction, mRNA-sequencing and proteome-wide proteomics studies

With glomeruli, part of them was used for total RNA extraction by using a kit (Qiagen, USA; 74134). The other part was used for protein extraction by using our recently published procedure (Chi *et al.* 2025*a*) after freeze at −80°C and thaw. Extractions of total RNA and protein from frozen cortices were as described (Chi *et al.* 2025*a*). Subsequent mRNA-sequencing and proteomics studies were conducted as described (Chi *et al.* 2025*a*).

### Measurements of triglycerides (TG) and total cholesterol (TC) in tissues

10–50 mg of frozen kidney cortices or liver tissues were used for lipid extraction (Triglyceride or Cholesterol Assay Kit, Signosis, USA). TG and TC were measured according to EA-7010 and EA-7011, respectively.

### Statistics

Statistical analysis was performed using GraphPad Prism 10.4.2. Data are expressed as the mean ± SD. Statistical differences between two groups were analyzed by unpaired, two-tailed Student’s *t*-test. A *P* value of less than 0.05 was considered statistically significant.

## Results

### Retinoic acid receptors are differentially altered in glomeruli from NS patients

**Figure 1:**
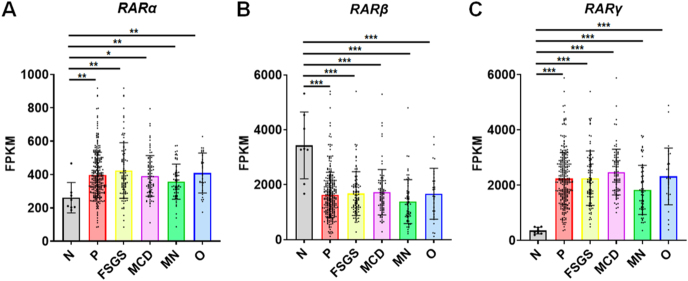
mRNA levels of *RARs* in kidney glomeruli are differentially changed in NS patients versus normal individuals. mRNA abundance was obtained from mRNA-sequencing of glomeruli of 8 normal individuals (N) and 274 patients (P) with nephrotic syndrome (NS) provided by the Nephrotic Syndrome Study Network (NEPTUNE) (GSE197307). NS was classified as focal segmental glomerulosclerosis (FSGS), MCD, MN, and others (O). P, all patients. FPKM, fragments per kilobase per million mapped fragments. *P ≤ 0.05, **P ≤ 0.01, ***P ≤ 0.001. A full color version of this figure is available at https://doi.org/10.1530/JME-25-0146.

RA treatment can prevent podocyte apoptosis and reduce proteinuria in murine models of NS (Suzuki *et al.* 2003, He *et al.* 2007, Chen *et al.* 2021). To better understand the molecular mechanisms by which RA exerts its beneficial effects, we searched and obtained a public database for molecular changes in NS patients (the Nephrotic Syndrome Study Network (NEPTUNE)) in Gene Expression Omnibus, GSE197307 (Fan *et al.* 2025). Figure 1 shows the analysis of *Rar* mRNA in glomeruli of normal individuals and NS patients. In normal individuals, *Rarβ* mRNA is the most abundant of the three *RARs*. Its expression level is about ten-fold higher than either *RARα* or *RARγ*. *RARβ* mRNA is reduced by 50% in NS patients (Fig. 1B). NS patients were classified into four categories: focal segmental glomerulosclerosis (FSGS), MCD, MN, and others (O). *Rarβ* is reduced in all four categories. In contrast to the decreases in *Rarβ*, *Rarα* mRNAs increased by about 50% in NS patients compared to normal individuals (Fig. 1A) and *RARγ* mRNAs increased by more than eight-fold (Fig. 1C). These data demonstrate differential changes in *Rar* mRNA in human NS. To test whether reduced *Rarβ* function could contribute to the development of NS, we generated a mouse model with *Rarβ* specifically deleted in podocytes, since podocyte injury is closely associated with NS (Clark *et al.* 2022).

### Generation and verification of the murine model: podocyte-specific deletion of *Rarβ*

Podocytes are the most abundant cell type in glomeruli (up to 80% of cells) (Karaiskos *et al.* 2018), and podocin is the most abundant protein in podocytes (Ruotsalainen *et al.* 1999). The Nphs2 (podocin) gene is first expressed at ∼embryonic day 16.5 in mice. *Rarβ* mRNA is expressed in podocytes (Gerhardt *et al.* 2023, Muto *et al.* 2024). To create a mouse model in which *Rarβ* is deleted specifically in the podocytes of glomeruli, we crossed the transgenic mouse line (PCR) expressing Cre under an Nphs2 (podocin) promoter (Pod-Cre) (Moeller *et al.* 2003) with floxed *Rarβ* mice (*Rarβ*^fl/fl^); this cross resulted in a mouse line with *Rarβ* specifically deleted in podocytes (PCRB) (Supplementary Fig. 1A). To verify this mouse model, we extracted DNA from primary glomeruli isolated from kidney cortices and livers (as a negative control) of PCRB and WT mice, and using genotyping PCR, we detected the expression of PodCre sequences in both glomeruli and livers of PCRB, but not WT mice (Supplementary Fig. 1B). We detected the *Rarβ* allele in WT and the *Rarβ* alleles with loxP sites only in PCRB mice. Importantly, the excised ‘floxed’ DNA fragment is present only in purified glomeruli, but not in the livers of the PCRB mice (Supplementary Fig. 1B), indicating deletion of the *Rarβ* gene. To further confirm the loss of *Rarβ expression* in podocytes, we conducted quantitative RT-PCR (qRT-PCR) using total RNA extracted from glomeruli. *Rarβ* mRNA in PCRB glomeruli is reduced by 80% compared to that in WT glomeruli (Supplementary Fig. 1C). The residual *Rarβ* mRNA in PCRB glomeruli is likely from other types of cells that constitute 20% of glomeruli (Karaiskos *et al.* 2018). These data demonstrate that *Rarβ* was specifically deleted in podocytes.

### Podocyte-specific *Rarβ* deletion results in albuminuria

**Figure 2:**
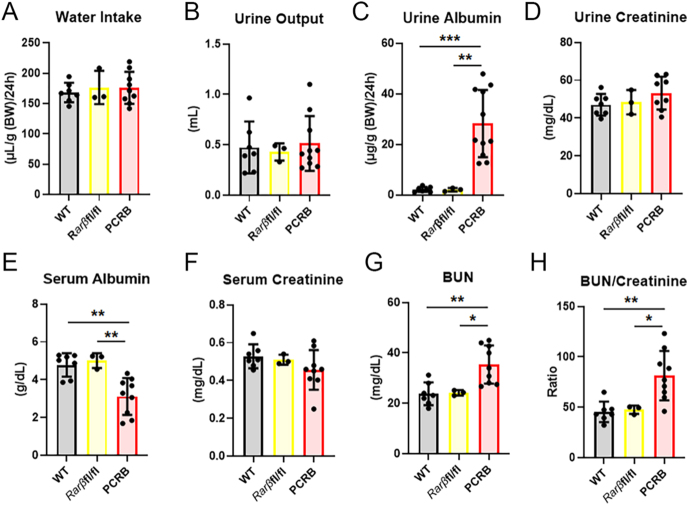
Deletion of *Rarβ* in podocytes results in dysfunctional kidneys and albuminuria in 5–6-month-old mice. Water intake and urine output for 24 h were recorded. 24 h urine was collected for measurements of urine albumin and creatinine. Blood was collected, and serum was separated from blood cells. Markers of renal functions in both urine and serum were measured by spectrometry. WT, wild type; *Rarβ*^fl/fl^, *Rarβ* floxed; PCRB, pod/cre; *Rarβ*. BUN, blood urea nitrogen. Each dot represents one mouse. Values = mean ± SD, *n* = 3–12 mice/group, *P ≤ 0.05, **P ≤ 0.01, ***P ≤ 0.001. A full color version of this figure is available at https://doi.org/10.1530/JME-25-0146.

After generation and verification of mice with *Rarβ* deleted specifically in the podocytes, we first assessed whether this deletion had effects on kidney function. We put three groups of mice (WT, *Rarβ*^fl/fl^, and PCRB) in metabolic cages and found no differences in either water intake or 24 h urine output among the groups (Fig. 2A and B). Further analyses of metabolites in urine and serum showed that urine albumin, a strong indicator of albuminuria, was almost 14-fold higher in PCRB than in WT (Fig. 2C). The increase in urine albumin was accompanied by reduced albumin in serum (Fig. 2E).

Another marker of kidney filtration ability is creatinine disposal. Urine or serum creatinine was not significantly different among the groups (Fig. 2D and F). However, blood urea nitrogen (BUN) and the ratio of BUN to creatinine were about 50–100% higher in PCRB than in WT (Fig. 2G and H). Overall, these markers indicate that the kidneys of PCRB mice are dysfunctional, i.e., expression of *Rarβ* is necessary for normal kidney function. For all of these markers, there were no differences between WT and *Rarβ*^fl/fl^ mice (Fig. 2), indicating that the kidney dysfunction in PCRB mice was caused by *Rarβ* deletion in podocytes. Therefore, for the following studies, we focused on comparing PCRB to WT mice.

### *Rarβ* deletion downregulates podocin expression, disrupts glomerular ultrastructure, and promotes lipid accumulation

**Figure 3:**
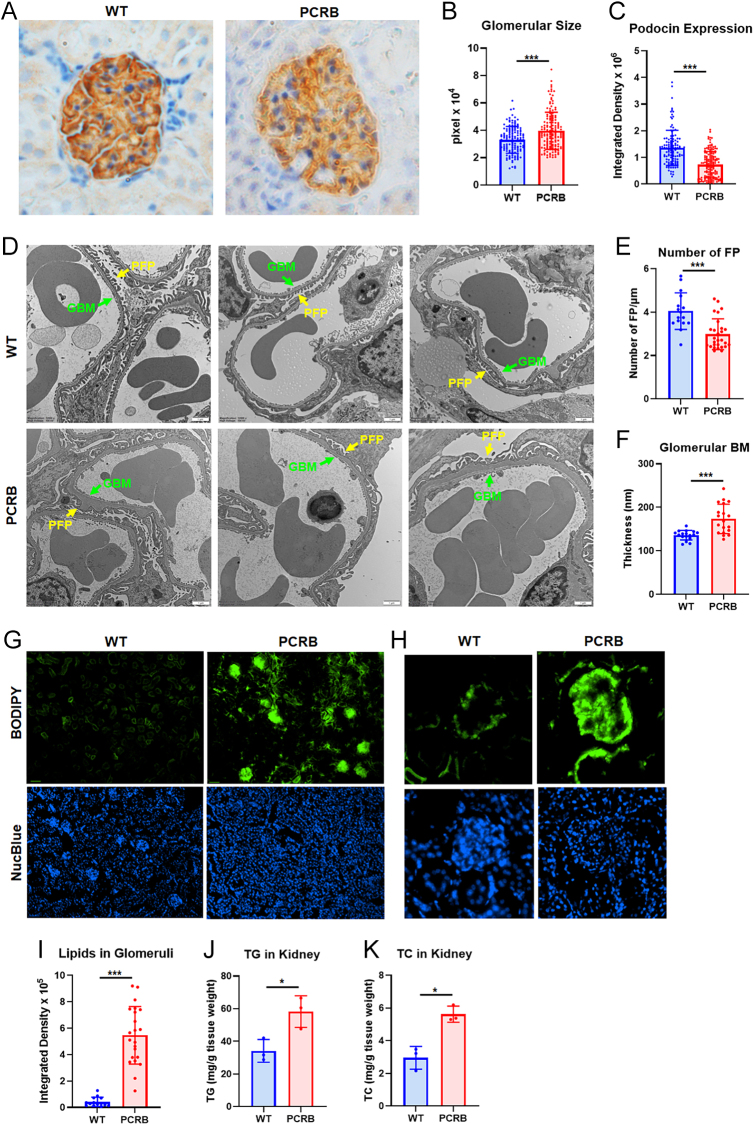
*Rarβ* deletion in podocytes impairs integrity of glomeruli and increases lipid deposition in glomeruli. (A) Kidney staining with podocin antibody; (B and C) quantitative analysis of glomerulus size (B) and podocin protein (C) by ImageJ; each dot in B and C represents one glomerulus from three WT and three PCRB mice, 40–50 glomeruli/mouse; (D) transmission electron microscopy (TEM) images of glomeruli from three WT and three PCRB mice; GBM, glomerular basement membrane; PFP, podocyte foot process; (E) quantitative analysis of number of foot processes (FP) per μm GBM, each dot represents one image from three WT and three PCRB mice, 7–9 images/mouse; (F) quantitative analysis of thickness of GBM, each dot represents one image from three WT and three PCRB mice, 5–7 images/mouse; (G) BODIPY staining for visualization of lipids in glomeruli; (H) zoomed-in BODIPY staining for visualization of lipids in one glomerulus. Lipids were marked by BODIPY, nuclei were marked by NucBlue; (I) quantitative analysis of lipid levels detected by BODIPY, each dot represents one field from three WT and three PCRB mice, 5–7 fields/mouse; (J and K) total triglycerides (TG) (J) and TC (K) in kidney cortices measured by respective kits from Signosis, *n* = 3 mice/group. Values = mean ± SD, *P ≤ 0.05, ***P ≤ 0.001. PFP, podocyte foot process. A full color version of this figure is available at https://doi.org/10.1530/JME-25-0146.

Normal glomeruli do not allow larger molecules, such as albumin, to be filtered out of blood and into urine. Appearance of albumin in the urine of PCRB mice indicates that the structures of PCRB glomeruli were likely compromised, resulting in protein leakage. Staining with *Nphs2* (podocin) (Franceschini *et al.* 2006) shows that PCRB glomeruli were enlarged by 17% (Fig. 3A and B), while podocin expression was reduced by 50% (Fig. 3C). Transmission electron microscopy (TEM) imaging reveals podocyte foot process (PFP) effacement (Fig. 3D, yellow arrows) and thickened glomerular basement membranes (GBM) in PCRB glomeruli (Fig. 3D, green arrows). Quantitative analyses show a significantly reduced number of PFPs (Fig. 3E) and increased GBM thickness (Fig. 3F). These structural changes likely impair the podocytes’ ability to prevent protein, such as albumin, from being filtered through glomeruli, leading to albuminuria (Fig. 2C). In addition to obvious morphological changes in PCRB glomeruli, we observed profound increases in lipid deposits in PCRB glomeruli, while the lipid level in WT glomeruli was minimal (Fig. 3G and H). Quantitative analysis shows the intensity of Bodipy (a marker of neutral lipids, such as TG) in PCRB glomeruli is more than seven-fold higher than that in WT glomeruli (Fig. 3I). We also measured TG in the kidney cortices. In PCRB cortices, TG was increased by 70% compared to WT cortices (Fig. 3J). In addition, TC was also increased in PCRB compared to WT cortices (Fig. 3K). Thus, the lack of *RARβ* signaling influences not only the structure of the kidney, but also metabolic processes in the kidney.

### Podocyte-specific *Rarβ* deletion impairs hepatic function and induces systemic hyperlipidemia

**Figure 4:**
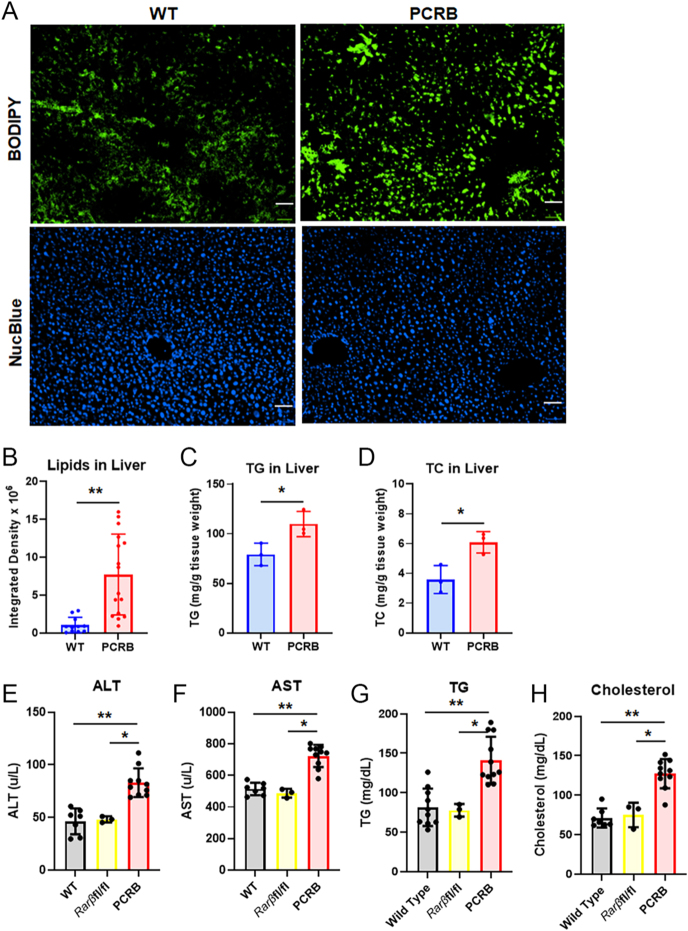
Deletion of *Rarβ* in podocytes increases lipid levels in liver, causes liver dysfunction, and systemic hyperlipidemia in 5–6-month-old mice. (A) BODIPY staining for visualization of lipids in liver. Lipids were marked by BODIPY, nuclei were marked by NucBlue; (B) quantitative analysis of lipid levels, each dot represents one field from three WT and three PCRB mice, 5–7 fields/mouse; (C and D) total triglycerides (TG) (C) and TC (D) in liver tissue measured by respective kits from Signosis, *n* = 3 mice/group; (E) ALT (alanine transaminase) and (F) AST (aspartate transaminase); (G and H) levels of triglycerides (TG) and cholesterol in blood serum, each dot represents one mouse. Values = mean ± SD, *n* = 3–12 mice/group, *P ≤ 0.05, **P ≤ 0.01. WT, wild type; *Rarβ*^fl/fl^, *Rarβ* foxed; transgenic; PCRB, pod/cre; *Rarβ*. A full color version of this figure is available at https://doi.org/10.1530/JME-25-0146.

While harvesting tissues, we noticed fat deposits in PCRB livers. Further examination by Bodipy staining confirmed increased lipids in PCRB vs WT livers (Fig. 4A and B). Additional analyses of both TG and TC in the liver by colorimetry assays demonstrate that both TG and TC increased in PCRB compared to WT livers (Fig. 4C and D). Fatty liver is usually associated with liver dysfunction. Indeed, serum ALT in PCRB mice was almost two-fold higher than that in WT (Fig. 4E), and AST was increased by about a third (Fig. 4F). Furthermore, blood serum TG and cholesterol were doubled in PCRB compared to WT (Fig. 4G and H), indicating that PCRB mice display hyperlipidemia.

### Podocyte-specific *Rarβ* deletion increases glomerular transcripts involved in lipid synthesis and transport

**Figure 5:**
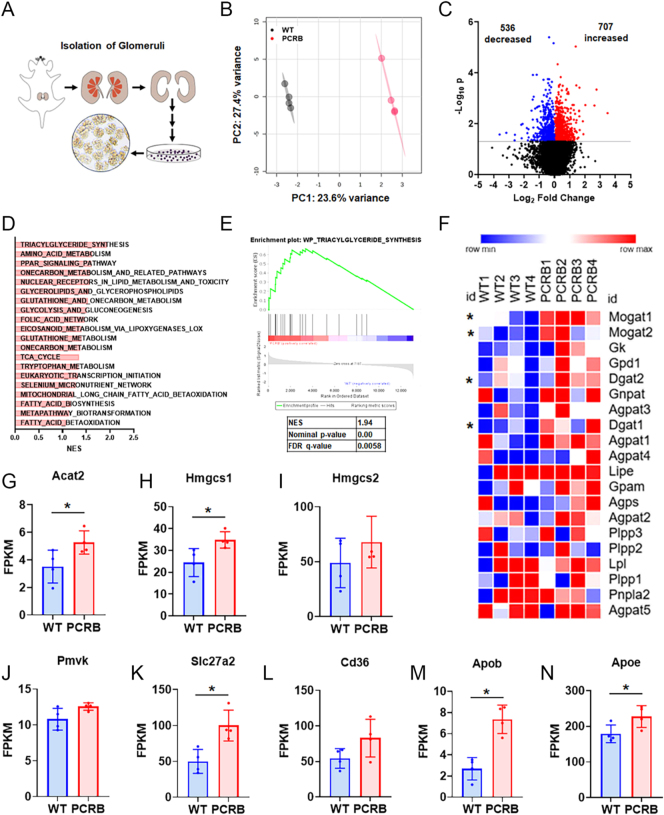
Deletion of *Rarβ* in podocytes caused genome-wide mRNA changes in glomeruli. (A) Diagram of isolation and purification of glomeruli; (B) principal component analysis (PCA) of transcripts for comparison of isolated, purified glomeruli between *Rarβ* KO (PCRB) vs WT; (C) volcano plots of −log_10_(p_adj_) versus log_2_(fold change) of gene expression in PCRB over WT. p_adj_ and log_2_(fold change) were calculated using R package DESeq2. Significantly decreased mRNAs are in blue and significantly increased mRNAs are in red; (D) top 20 enriched gene sets obtained by Gene Set Enrichment Analysis (GSEA); (E) plot of enriched gene set in triglyceride (TG) synthesis; (F) heatmap of genes involved in TG synthesis, 4 mice/group; (G, H, I, J, K, L, M) individual genes participating in cholesterol synthesis, fatty acid/lipid uptake, and lipid efflux. Note that the changes in glomerular mRNAs in PCRB vs WT could reflect changes in podocytes themselves and/or in mesangial and endothelial cells, which are also present in glomeruli. *P ≤ 0.05. A full color version of this figure is available at https://doi.org/10.1530/JME-25-0146.

To explore the molecular mechanisms mediating these phenotypic abnormalities in PCRB mice, we conducted genome-wide mRNA sequencing of glomeruli isolated from cortices of both WT and PCRB mice (Wang *et al.* 2019) (illustrated in Fig. 5A); we were able to obtain >95% pure glomeruli, as visualized by microscopy, at a high yield. We then isolated total RNA and conducted mRNA-seq. Principal component analysis (PCA) shows that the transcripts of WT and PCRB glomeruli are closely clustered but well separated (Fig. 5B), indicating that *Rarβ* deletion in podocytes caused a genome-wide shift in gene expression. Based on the criterion of fragments per kilobase per million mapped fragments (FPKMs) ≥ 1, about 13,200 transcripts were analyzed. *Rarβ* deletion in podocytes resulted in ∼10% of these transcripts being significantly changed, either induced or reduced (Fig. 5C). Gene set enrichment analysis (GSEA) shows that glycerolipids and TG synthesis were among the top upregulated gene sets in PCRB compared to WT (Fig. 5D and E). Increased transcripts in the TG synthesis gene set included *Mogat1* and *2,* and *Dgat1* and *2* (Fig. 5F). In addition, some mRNAs participating in cholesterol synthesis were increased (Fig. 5G, H, I). Besides mRNAs involved in lipid synthesis, mRNAs in lipid transport were affected by podocyte *Rarβ* deletion. There are mainly two groups of mediators that import lipids into cells: the SLC27A family transporters and the CD36 translocator. *Slc27a2*, the most abundantly expressed member of the *Slc27a* family in the kidney (Anderson & Stahl 2013), was significantly increased in PCRB compared to WT glomeruli (Fig. 5K), while *Cd36* trended higher (Fig. 5L). The lipid efflux mediators, including *ApoB* and *ApoE*, were also significantly increased in PCRB compared to WT glomeruli (Fig. 5M and N), potentially exporting lipids to the circulation.

### *Rarβ* deletion in podocytes upregulates glomerular proteins mediating lipid synthesis and uptake

**Figure 6:**
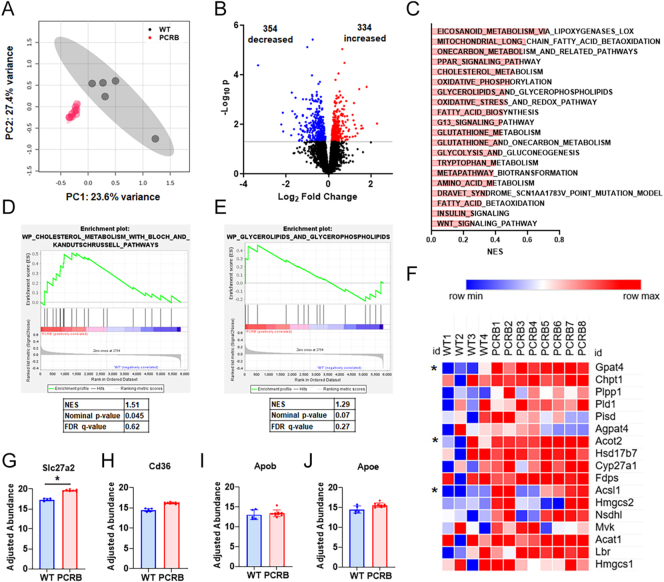
Deletion of *Rarβ* in podocytes causes proteome-wide changes in proteins of purified glomeruli. (A) Principal component analysis (PCA) of proteins for comparison between *Rarβ* KO (PCRB) vs WT; (B) volcano plots of −log_10_(p_adj_) versus log_2_(fold change) of protein levels in glomeruli of PCRB over WT. p_adj_ and log_2_(fold change) were calculated using R package DESeq2. Significantly decreased proteins are in blue and significantly increased proteins are in red; (C) top 20 enriched protein sets obtained by Gene Set Enrichment Analysis (GSEA); (D and E) plot of enriched protein set in synthesis of cholesterol (D) and glycerolipids (E and F) heatmap of proteins involved in cholesterol synthesis (four WT mice, and PCRB 1–8 were duplicated profilings of four PCRB mice); (G, H, I, J) individual proteins mediating fatty acid/lipid uptake (G and H) and lipid efflux (I and J).*P ≤ 0.05. A full color version of this figure is available at https://doi.org/10.1530/JME-25-0146.

To investigate whether the mRNA changes led to changes in protein levels, we conducted proteome-wide proteomics analyses of proteins extracted from the same glomeruli isolation that was used for total RNA extraction. PCA shows a separation in protein profiles between WT and PCRB (Fig. 6A). About 15% of the total number of proteins changed, with half of them increased and half of them decreased in PCRB compared to WT (Fig. 6B). Similar to the mRNA-seq data, synthesis of fatty acids, cholesterol, and glycerol lipids are among the top 20 upregulated protein sets (obtained by GSEA) (Fig. 6C, D, E). Increases in specific proteins in the cholesterol synthesis pathway are shown (Fig. 6F). Transporters (SLC27A2 and CD36) mediating fatty acid uptake were also increased in the PCRB glomeruli, although the increase in CD36 was moderate (Fig. 6G and H). However, APOB and APOE, proteins mediating lipid efflux, were not altered (Fig. 6I and J). Thus, both mRNA-seq and proteomics data demonstrate that both lipid synthesis and lipid uptake are upregulated in PCRB versus WT glomeruli. Increased production and uptake of fatty acids and lipids resulted in greater lipid levels in PCRB vs WT glomeruli (Fig. 3G, H, I, J, K).

### mRNAs in fatty acid and lipid synthesis are upregulated in human NS

**Figure 7:**
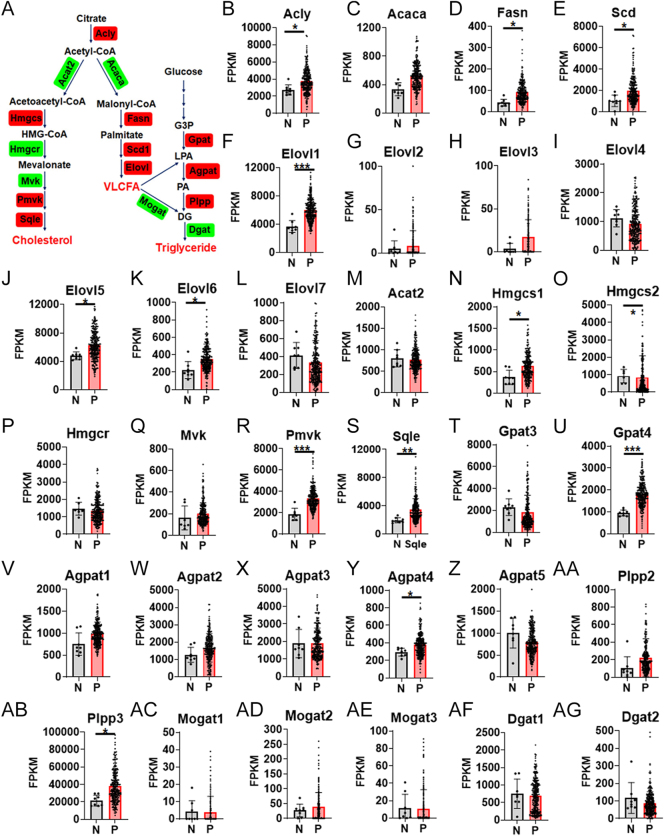
mRNAs involved in the synthesis of fatty acids and lipids in glomeruli were upregulated in NS patients. (A) Diagram of the synthesis of fatty acid, triglyceride, and cholesterol. Transcripts in red were increased in NS patients compared to normal individuals. Transcripts in green were not significantly changed. B-AG, mRNA counts of individual genes obtained from mRNA-sequencing of glomeruli of 8 normal individuals (N) and 274 patients (P) with NS provided by Nephrotic Syndrome Study Network (NEPTUNE) (GSE197307). VLCFA, very long chain fatty acid. Values = mean ± SD, *P ≤ 0.05, **P ≤ 0.01, ***P ≤ 0.001. A full color version of this figure is available at https://doi.org/10.1530/JME-25-0146.

To determine whether the upregulated transcripts and proteins for fatty acid and lipid synthesis seen in our mouse model are reflected in transcript changes in NS patients, we analyzed mRNA counts of all genes in fatty acid, TG, and cholesterol biosynthesis pathways (Fig. 7A). All transcripts in fatty acid synthesis, except *ACACA,* were increased by 30–500% in glomeruli from NS patients compared to normal individuals (Fig. 7B, C, D, E, F, G, H, I, J, K, L, M). In the cholesterol synthesis pathway, *HMGCS1* mRNA increased by two-fold (Fig. 7N), while *HMGCS2* was reduced (Fig. 7O). Besides changes in *HMGCS*, transcripts for both *PMVK* and *SQLE* were increased by almost two-fold in NS patients vs normal individuals (Fig. 7R and S). In the TG synthesis pathway, *GPAT4*, *AGPAT4,* and *PLPP3* transcripts were significantly increased in glomeruli from NS patients (Fig. 7U, Y, AB). Overall, most transcripts in all three pathways were increased in NS patients. These data, along with data in Fig. 5, indicate that many mRNAs in the fatty acid and lipid biosynthesis pathways are similarly upregulated in glomeruli of both NS patients and the PCRB mice. Thus, reduced *RARβ* may be one of the factors that contributes to the pathological features, such as lipotoxicity, in NS patients.

### Podocyte-specific *Rarβ* deletion also increases hepatic transcripts involved in cholesterol biosynthesis and lipid uptake pathways

**Figure 8:**
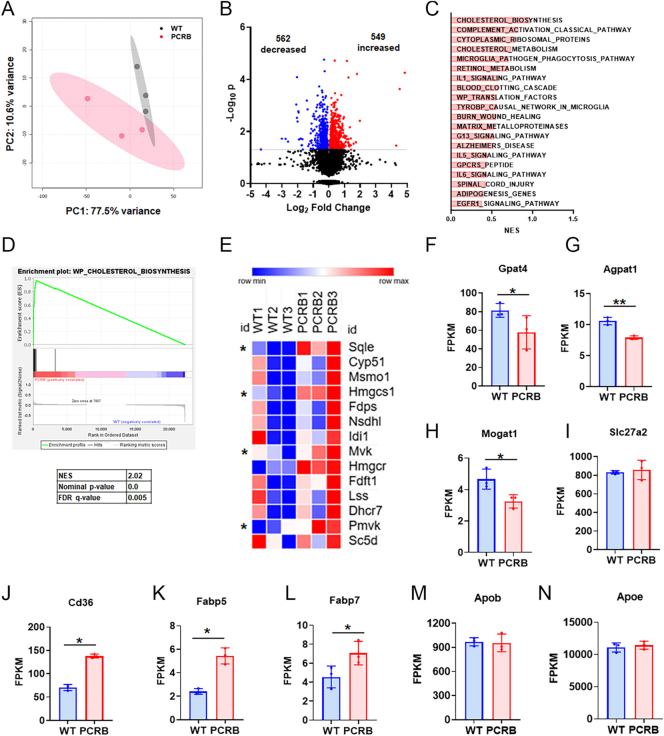
Deletion of *Rarβ* in podocytes upregulates mRNAs in the synthesis and uptake of fatty acids/lipids in liver. (A) Principal component analysis (PCA) of transcripts for comparison between *Rarβ* KO (PCRB) vs WT; (B) volcano plots of −log_10_(p_adj_) versus log_2_(fold change) of gene expression in PCRB over WT. p_adj_ and log_2_(fold change) were calculated using R package DESeq2. Significantly decreased transcripts are in blue and significantly increased transcripts are in red; (C) top 20 enriched gene sets obtained by Gene Set Enrichment Analysis (GSEA); (D) plot of enriched gene set in cholesterol synthesis; (E) heatmap of genes involved in cholesterol synthesis; (F, G, H) individual genes participating in TG synthesis; (I, J, K, L, M, N) individual genes mediating fatty acid/lipid uptake (I and J), fatty acid translocation (K and L), and lipid efflux (M and N). *P ≤ 0.05, **P ≤ 0.01. A full color version of this figure is available at https://doi.org/10.1530/JME-25-0146.

As mentioned above, *Rarβ* deletion in podocytes unexpectedly caused lipid/cholesterol deposits in PCRB livers (Fig. 4A and B). We therefore assessed changes in transcripts genome-wide in WT and PCRB livers. Greater than 77% of the transcripts were separated between these two groups by PCA (Fig. 8A). In liver, we detected 10,882 transcripts with FPKMs ≥ 1, of which 10% were changed significantly (Fig. 8B). GSEA shows that cholesterol biosynthesis is the top gene set upregulated in the PCRB livers (Fig. 8C and D). Most transcripts in the cholesterol synthesis pathway (*Hmgcs, Mvk, Pmvk,* and *Sqle*) were increased in PCRB vs WT livers (Fig. 8E). In contrast, transcripts involved in TG synthesis, including *Gpat, Agpat,* and *Mogat*, were reduced in PCRB compared to WT livers (Fig. 8F, G, H), suggesting that the increased TGs seen in PCRB livers (Fig. 4) were not due to *de novo* lipid synthesis in the liver. We then found that the transcript for *Cd36*, which mediates fatty acid and lipid uptake from the circulation (He *et al.* 2011), increased by > two-fold in PCRB vs WT livers (Fig. 8J); this could potentially indicate import of more fatty acids for increased cholesterol synthesis. *Fabp5* and *Fabp7* transcripts were also increased in PCRB livers (Fig. 8K and L), potentially accelerating translocation of fatty acids within cells. The lipid efflux mediators, *ApoB/E*, did not change in PCRB vs WT livers (Fig. 8M and N).

### Podocyte-specific *Rarβ* deletion upregulates hepatic proteins mediating both fatty acid/cholesterol biosynthesis and uptake in the liver

**Figure 9:**
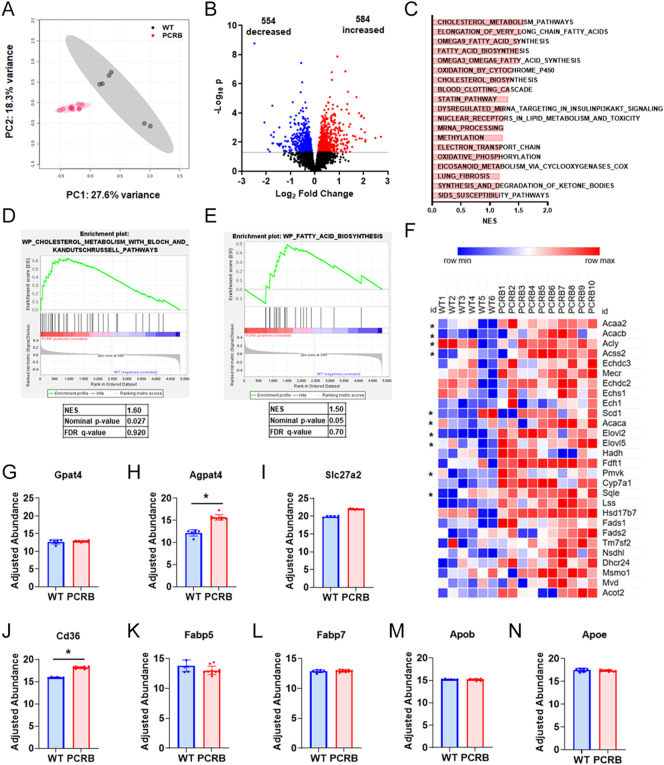
Deletion of *Rarβ* in podocytes increases levels of proteins in the synthesis and uptake of fatty acids and lipids in liver. (A) Principal component analysis (PCA) of proteins for comparison between *Rarβ* KO (PCRB) vs WT; (B) volcano plots of −log_10_(p_adj_) versus log_2_(fold change) of protein expression in PCRB over WT. p_adj_ and log_2_(fold change) were calculated using R package DESeq2. Significantly decreased proteins are in blue and significantly increased proteins are in red; (C) top 20 enriched protein sets obtained by Gene Set Enrichment Analysis (GSEA); (D and E) plot of enriched protein sets in synthesis of cholesterol (D) and fatty acids (E and F) heatmap of proteins involved in the synthesis of fatty acids and cholesterol; (G and H) individual genes participating in TG synthesis; (I, J, K, L, M, N) individual proteins mediating fatty acid/lipid uptake (I and J), fatty acid translocation (K and L), and lipid efflux (M and N). WT 1–6 were duplicated profilings of three WT mice; PCRB 1–10 were duplicated profilings of five PCRB mice. *P ≤ 0.05. A full color version of this figure is available at https://doi.org/10.1530/JME-25-0146.

We further conducted proteome-wide proteomics studies on proteins extracted from livers. Similar to the mRNA-seq data, PCA shows that *Rarβ* deletion in podocytes changes protein profiles in the liver (Fig. 9A). Out of 4,828 detected proteins, about 20% were significantly changed (Fig. 9B). The top 20 upregulated protein sets (generated by GSEA) in PCRB livers included not only cholesterol synthesis, but also fatty acid synthesis (Fig. 9C, D, E), which is different from the mRNA-seq data (Fig. 8C), suggesting regulation at a different level. Almost all proteins in the fatty acid synthesis pathway were increased in PCRB livers (Fig. 9F). In addition, one enzyme, AGPAT4, involved in TG synthesis, was also increased in PCRB livers (Fig. 9H). Consistent with the mRNA-seq data, CD36 protein was significantly increased in PCRB vs WT livers (Fig. 9J), while SLC27A2 was not significantly changed (Fig. 9I). Increased CD36 protein levels in PCRB compared to WT livers were confirmed by IHC (Supplementary Fig. 2). Also, consistent with the mRNA-seq data, the proteins mediating lipid efflux did not change in WT vs PCRB livers (Fig. 9M and N). Taken together, the mRNA-seq and proteomics data strongly indicate that *Rarβ* deletion in podocytes causes increased uptake and synthesis of fatty acids and cholesterol in the liver, which likely contributes to systemic hyperlipidemia.

Hyperlipidemia is often linked to abnormal glucose metabolism (Parhofer 2015), so we conducted glucose tolerance tests (Supplementary Fig. 3A). The higher area under the curve (AUC) in PCRB compared to WT mice (Supplementary Fig. 3B) indicated that PCRB mice were glucose intolerant. Thus, *Rarβ* deletion in podocytes leads not only to hyperlipidemia, but also to glucose intolerance.

## Discussion

As mentioned before, NS is divided into 3–4 main pathophysiological subtypes: MCD, FSGS, MN, and others (O) (Verma & Patil 2024). For all these types of NS, the podocyte is thought to be the primary target of cellular injury in NS (Ding & Saleem 2012). Reduced podocyte density and altered podocyte morphology are directly linked to proteinuria/albuminuria (Shankland 2006). The main protein expressed in podocytes is podocin (Nphs2), which is a major marker of podocytes (Franceschini *et al.* 2006). In PCRB mice, NPHS2 expression was reduced (Fig. 3C), and podocyte foot processes were deformed (Fig. 3D), leading to albuminuria (Fig. 2C) and hypoalbuminemia (Fig. 2E). These phenotypic features in PCRB mice mimic the clinical features of NS (Politano *et al.* 2020), suggesting that PCRB mice are a valuable tool for investigation of molecular and possibly cellular mechanisms of NS, particularly the MCD type of NS. The majority of MCD is idiopathic. This study provides a possible cause of MCD: low expression or loss of *Rarβ*.

NS is one of the major causes of both end-stage kidney disease (Verma & Patil 2024) and acute renal failure (Koomans 2001). Despite the severity of NS, the currently available treatments are limited. Glucocorticoids and/or calcineurin inhibitors are the major therapies used for FSGS, MCD, and IMN (Politano *et al.* 2020). Angiotensin-converting enzyme inhibitors (ACEIs) and angiotensin receptor blockers (ARBs) are used when NS is accompanied by hypertension. For diabetic nephropathy, the strategies are glycemic control and/or blood pressure control with ACEIs or ARBs. Hyperlipidemia often co-exists with NS (Bobulescu 2010), and statins are used to treat the hyperlipidemia (Politano *et al.* 2020). Proprotein convertase subtilisin-kexin type 9 inhibitors are also used (Schelling 2022). However, none of these drugs was developed to specifically treat NS. Thus, drugs acting through *Rarβ* or on the pathways controlled by *Rarβ* may be useful therapeutics.

Retinoic acid (RA), a biologically active metabolite of vitamin A, regulates gene expression and biological functions by interacting with its three receptors (RARα, RARβ, and RARγ) (Gudas 2022*b*). We have shown that these receptors play important roles in maintaining the normal physiology of several tissues and cell types, including those in the kidney and liver (Melis *et al.* 2022, DiKun & Gudas 2023). For instance, *Rarα* deletion in kidney proximal tubules resulted in kidney injury and fibrosis (DiKun *et al.* 2024). In this study, we demonstrate that *Rarβ* is critical for maintaining the normal structure and functions of glomeruli, as *Rarβ* deletion in podocytes results in abnormal glomerular structure and function, lipid accumulation in glomeruli, and albuminuria (Figs. 2 and 3). *Rarβ* deletion in podocytes also results in increased fatty acid and lipid biosynthesis in the liver, liver dysfunction, and hyperlipidemia (Fig. 4). These data provide support for developing selective RAR agonists as drugs that would complement existing therapies for NS.

The podocin protein is intimately related to lipids, as it localizes to the cholesterol-concentrated lipid rafts (Schwarz *et al.* 2001). Reports of concurrence of albuminuria and lipid deposits in glomeruli of NS patients appeared as early as 1969 (Hayslett *et al.* 1969). Since the ‘lipid nephrotoxicity hypothesis’ proposed in 1982 (Moorhead *et al.* 1982), many studies have provided evidence suggesting that lipotoxicity in the kidney is the cause of albuminuria (Bobulescu 2010). Here, we show that most mRNAs in the fatty acid and lipid synthesis pathways are upregulated in glomeruli of NS patients (Fig. 7). Utilizing both transcriptomic and proteomics approaches, we found that, at the molecular level, the primary effect of *Rarβ* deletion in podocytes was the upregulation of fatty acid/lipid biosynthesis in glomeruli (Figs 5 and 6). These results also support the hypothesis that lipid accumulation and toxicity resulting from *Rarβ* deletion could be the cause of the deformed/damaged structure (Fig. 3) and the impaired function of glomeruli (Fig. 2).

**Figure 10:**
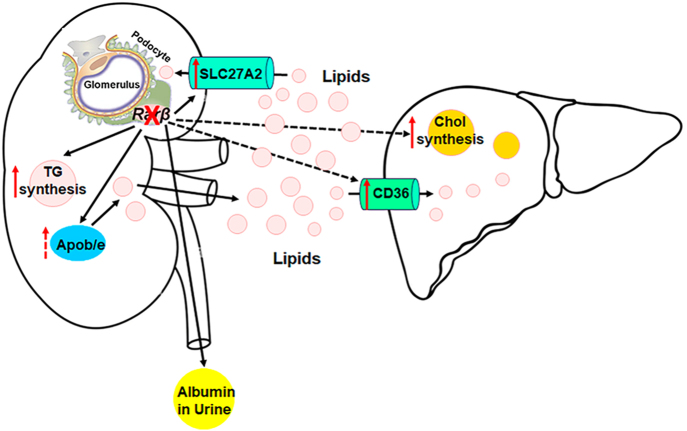
Proposed model of the consequences of loss of *Rarβ* in podocytes in both kidney and liver. *Rarβ* deletion in podocytes induces fatty acid uptake by SLC27A2 in glomeruli, increased triglyceride (TG) synthesis, and concomitantly causes albuminuria; *Rarβ* deletion also induces fatty acid uptake by CD36 and cholesterol synthesis in liver. Upregulated synthesis of fatty acids and lipids in both kidney and liver results in systemic hyperlipidemia. A full color version of this figure is available at https://doi.org/10.1530/JME-25-0146.

Lipid homeostasis is determined by several processes, including uptake of fatty acids and/or lipids, lipolysis and lipogenesis, and efflux of lipids or fatty acids. There are three groups of mediators for fatty acid transport: SLC27A family transporters, CD36, and FABPs (Schwenk *et al.* 2010, He *et al.* 2011, Anderson & Stahl 2013, Hotamisligil & Bernlohr 2015). The SLC27A family member is predominantly expressed in the kidney (Khan *et al.* 2018). At both the mRNA and protein levels, expression of SLC27A2 is consistently higher in PCRB than in WT glomeruli (Figs 5K and 6G), while CD36 trended higher (Figs 4K and 5H). In glomeruli of human NS patients, *CD36* mRNA was increased, while *SLC27A2* was decreased relative to levels in normal individuals (Supplementary Fig. 4). Higher levels of *CD36* could lead to the uptake of more fatty acids as precursors for increased TG synthesis (Fig. 10), as well as increased cholesterol synthesis (Fig. 10). Notably, CD36 was shown to aggravate podocyte injury via activation of the nod-like receptor family pyrin domain containing three inflammasome (Yang *et al.* 2017, Lv *et al.* 2022).

While lipid synthesis was increased by *RARβ* deletion, genes and proteins in lipolysis pathways were not significantly changed (Supplementary Fig. 5). In terms of lipid export, APOB is the principal structural protein in TG-rich lipoproteins secreted from the kidney (Barutta *et al.* 2022). Along with other APOs, such as APOE, APOB wraps around TGs and cholesterol and forms lipoproteins that carry TGs and cholesterol into cells (Noels *et al.* 2021). *Rarβ* deletion causes an increase in *ApoB* and *ApoE* transcripts (Fig. 5L and M), but not proteins (Fig. 6I and J). Overall, the net effect of podocyte *Rarβ* deletion on these four processes (import, export, production, and consumption) is an increase in lipids in glomeruli (Fig. 3F, G, H).

Besides causing dyslipidemia in the kidney and albuminuria, unexpectedly, we discovered that *Rarβ* deletion in podocytes causes an increase in lipids in the liver, liver dysfunction, and overall systemic hyperlipidemia (Fig. 4), suggesting either that RARβ in podocytes regulates signaling between the kidney and the liver or that a dysfunctional kidney releases lipids that are then stored by the liver. Numerous studies have demonstrated cross-signaling between the kidney and liver under physiological, and especially under pathological conditions (Capalbo *et al.* 2019, Sharma *et al.* 2022, Rad *et al.* 2024). Increased hepatic enzymes for cholesterol synthesis at both mRNA and protein levels have been reported in animal models of NS (Vaziri & Liang 1995, Vaziri *et al.* 2003, Vaziri *et al.* 2004, Zhou *et al.* 2008). Here, we show that, in the livers of mice with NS resulting from *Rarβ* deletion in podocytes, almost all proteins in the fatty acid synthesis pathway were elevated (Fig. 9F). Several enzymes in the synthesis pathways for triglycerides and cholesterol were also markedly increased in livers of PCRB mice (Figs 8B, C, D, E, F, G, H, I and 9B, C, D, E, F, G, H). CD36 is a major mediator of signaling between organs (Vaziri 2016, Yang *et al.* 2022). Elevated CD36 in both glomeruli and livers as a result of *Rarβ* deletion in podocytes may contribute to the cross-signaling between kidney and liver. However, delineating how *Rarβ* deletion in podocytes affects the levels of various mRNAs and proteins in the liver requires further investigation. This research provides evidence that RARβ expression, and perhaps activation, can reduce lipid uptake by podocytes, which can cause podocyte injury, glomerular deformation, and glomerular dysfunction.

## Supplementary materials



## Declaration of interest

There is no conflict of interest that could be perceived as prejudicing the impartiality of the research.

## Funding

This work was supported by W81XWH-22-1-0873, NIH R01 DK113088, 5T32CA062948, and Weill Cornell Medicine Genitourinary Oncology Research Fund.

## Author contribution statement

LJG and YC conceived of the project, designed and conducted experiments, wrote the original draft, and edited the revision. KMD, CDW, and JBG conducted experiments and analyzed data. XHT conducted experiments and edited the manuscript. SC and EMDA conducted experiments. JAW provided advice and edited the manuscript.

## Data availability

We have deposited mRNA-seq data into Gene Expression Omnibus. Proteomics data are deposited into MassIVE, accession # MSV000097819. All raw data are available upon request from the corresponding author.
