# Supplementary material for: Retinoic acid receptor β deletion in podocytes causes kidney and liver dysfunction, modeling nephrotic syndrome
Source: J Mol Endocrinol. 2026 Jan 2;76(1):e250146. doi: 10.1530/JME-25-0146 (PMC12822819; doi:10.1530/JME-25-0146)
Supplement: Supplementary file 1 [file supplementary_materials.pdf]

Materials and Methods

Acquisition and analysis of mRNA-seq data from NS patients and normal individuals. We acquired database (GSE197307) of Glomerular Transcriptome for the Nephrotic Syndrome Study Network (NEPTUNE) cohort from The National Center for Biotechnology Information (NCBI). In this study, 8 normal individuals and 274 patients were enrolled. Those 274 patients were classified into 4 groups based on the type of NS, focal segmental glomerulosclerosis (FSGS), minimal change disease (MCD), membranous nephropathy (MN), and others (O). There were 93 FSGS patients, 89 MCD patients, 62 MN patients, 30 others. We downloaded genome wide RNA-seq counts of each enrolled person. All gene counts of all enrolled people and analysis output are presented in the Excel file, which is in Supplementary Materials. We grouped them into N (normal individual), FSGS, MCD, MN, O, and P (all patients). For each gene of interest, we plotted mRNA counts and analyzed mean value and significance (p value) using Prism program.

Results

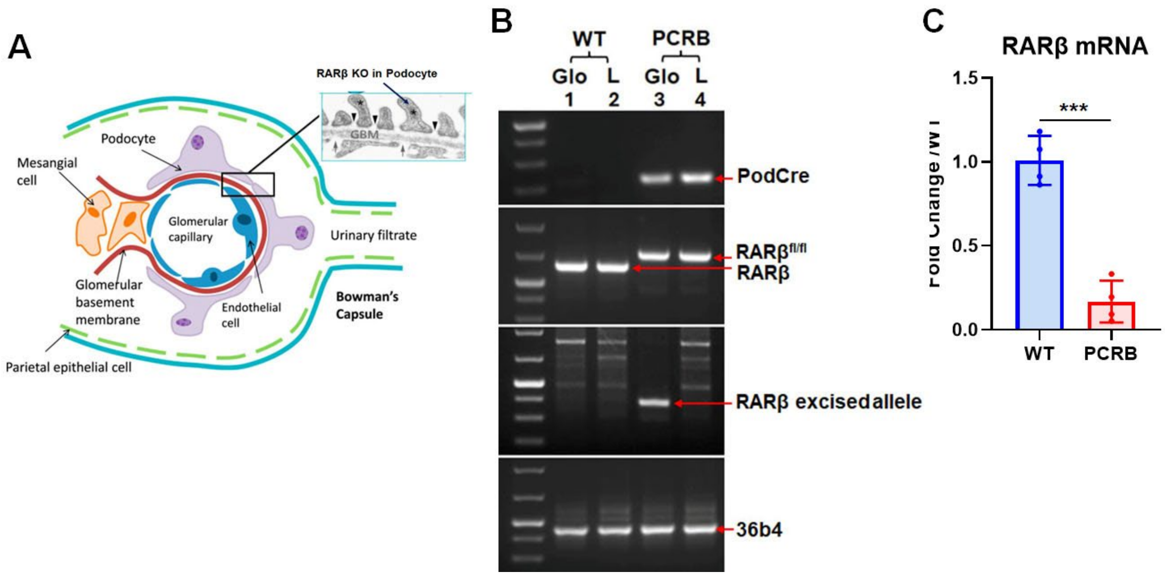

Supplementary Figure 1. A, schematic depiction of *Rarβ* deletion in podocytes in glomerulus; B, verification of specific deletion of *Rarβ* in podocytes by genotyping with DNA extracted from glomeruli, or liver (as negative reference); PCRs were run as described in the Methods section; Glo, glomeruli; L, liver. 36b4 is a loading control. The *Rarβ* gene is modified in the glomeruli, but not in the liver. C, *Rarβ* mRNA measured by qRT-PCR of total RNA extracted from glomeruli, each dot represents isolated glomeruli from one mouse, 4 mice/group. Values = mean ± SD, ***$p \leq 0.001$. WT, wild type; PCRB, pod/cre;*Rarβ*.

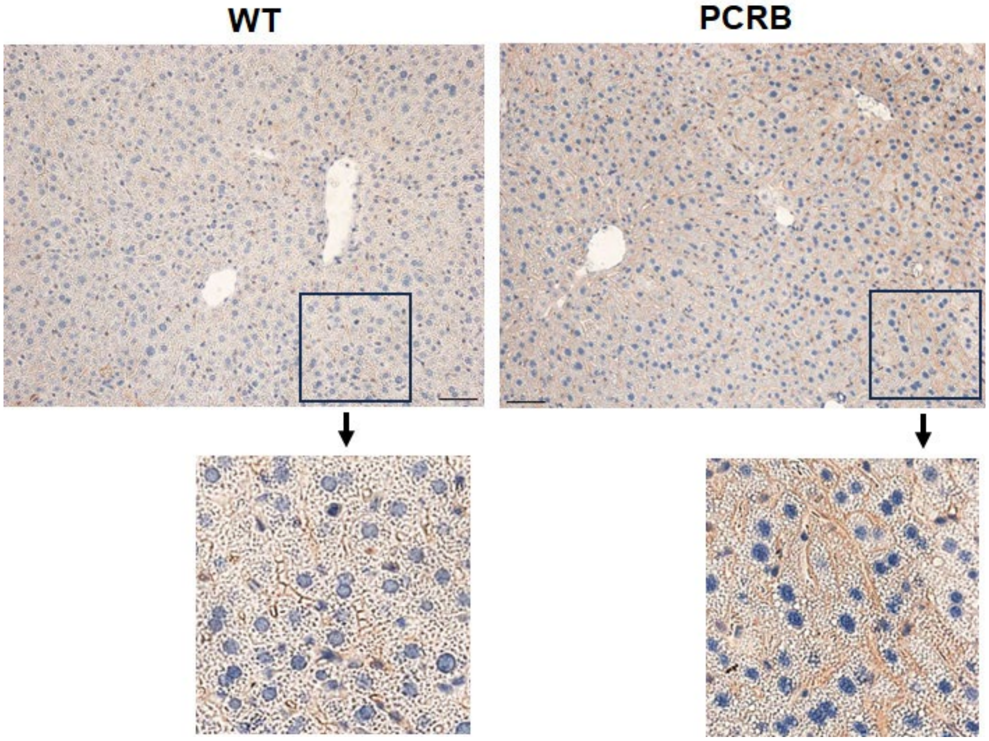

Supplementary Figure 2. Deletion of *Rarβ* increased CD36 in liver. CD36 staining of liver tissues from WT and PCRB mice.

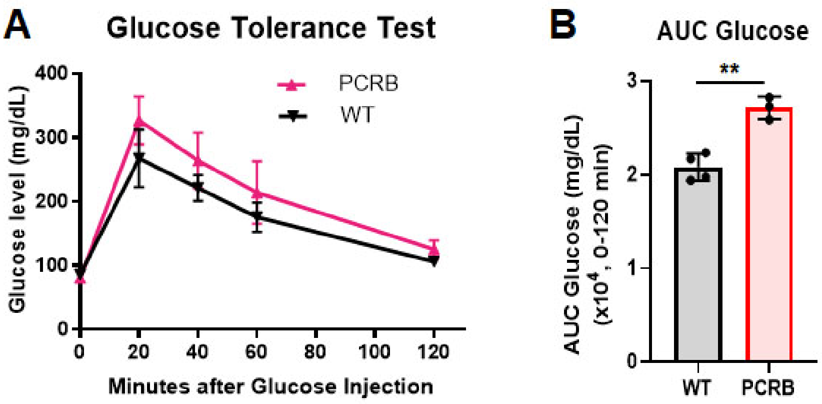

Supplementary Figure 3. Deletion of *Rarβ* in podocytes results in glucose intolerance. Values = mean ± SD, **p ≤ 0.01.

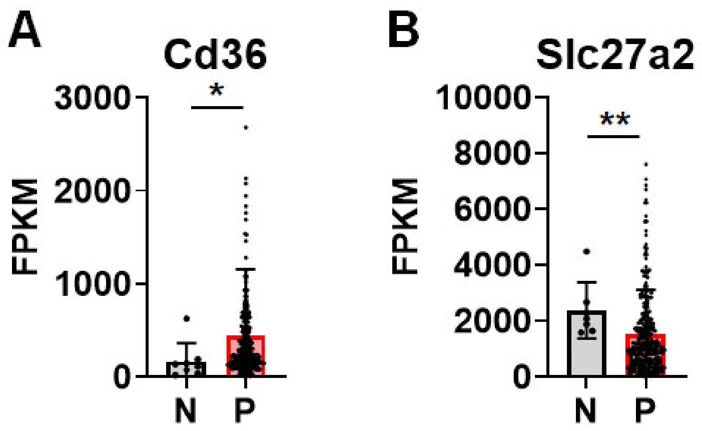

Supplementary Figure 4. mRNA counts of *CD36* and *SLC27A2* obtained from mRNA-sequencing of glomeruli of 8 normal individuals (N) and 274 patients (P) with NS provided by Nephrotic Syndrome Study Network (NEPTUNE) (GSE197307). Values = mean ± SD, *p ≤ 0.05, **p ≤ 0.01.

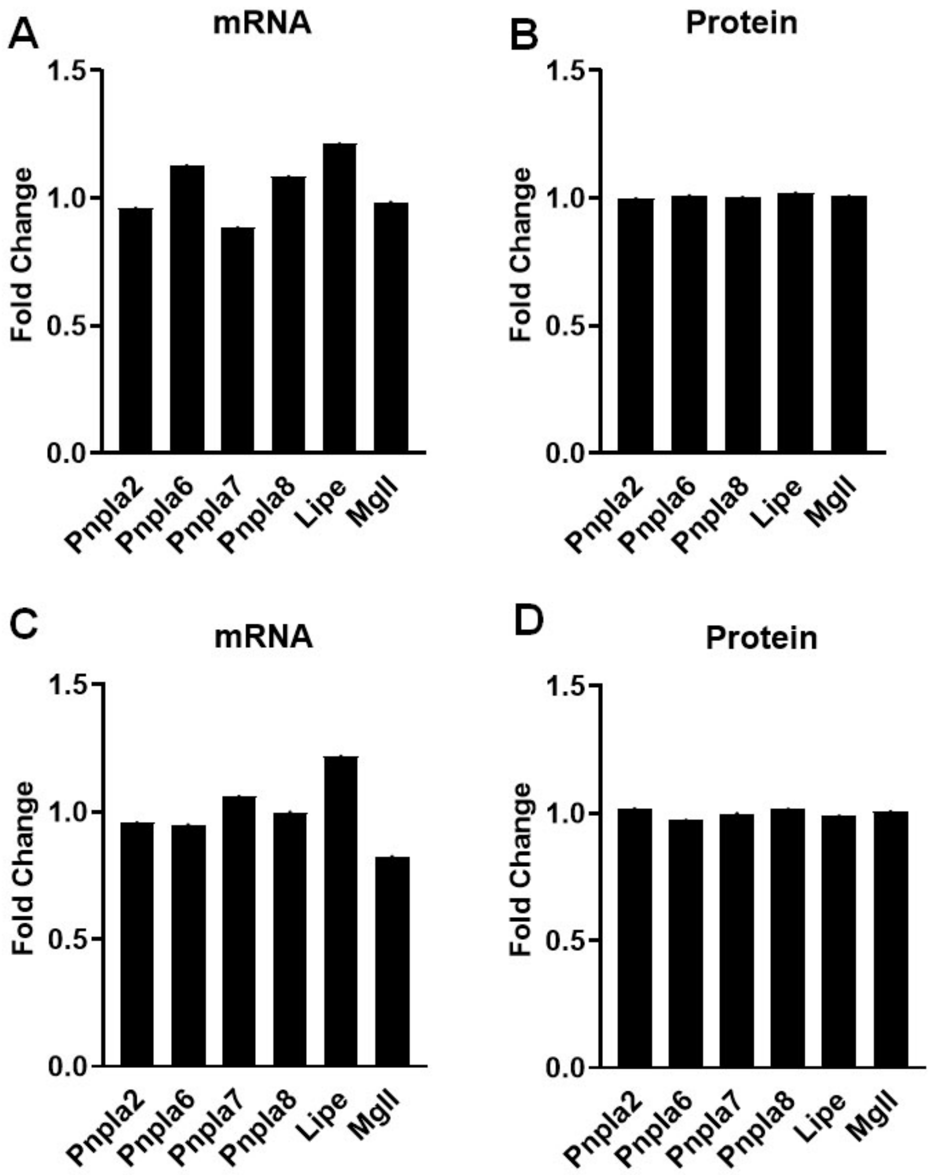

Supplementary Figure 5. Deletion of *Rarβ* in podocytes does not affect lipolysis in either glomeruli or liver. A, B, fold changes of mRNAs (A) and proteins (B) participating in lipolysis in PCRB glomeruli over WT glomeruli; C, D, fold changes of mRNAs (C) and proteins (D) participating in lipolysis in PCRB liver over WT liver.

Supplementary Table 1. Primers for genotyping

Gene	Forward Primer	Reverse Primer
PodCre	5'GCGCTGCTGCTCCCAG3'	5'CGGTTATTCAACTTGCACCA3'
Rarβ or *Rarβ* $^{fl/fl}$	5'TGTACCCAGAGTCAACAAA3'	5'GGCCATCAGAGAAAGTCAT3'
Excised allele	5'TGTACCCAGAGTCAACAAA3'	5'CAACCCAGTCCAGCACCAG3'
36B4	5'AGAACAACCCAGCTCTGGAGAAA3'	5'ACACCCTCCAGAAAGCGAGAGT3'

Supplementary Table 2. List of antibodies

Antibody	Source (Catalogue #)	Dilution
Podocin (NPHS2)	Abcam (Ab181143)	1:400
CD36	Cell Signaling (14347S)	1:100
